# Managing Bipolar Disease Complicated with Psychosis in Conjunction with Polypharmacy, Parkinson's Disease, and Multiple Comorbidities

**DOI:** 10.1155/2022/3813929

**Published:** 2022-05-05

**Authors:** Ricardo Irizarry, Ariel Sosa Gomez, Simeon Miles, Jean Tamayo Acosta

**Affiliations:** ^1^South Texas Health System, USA; ^2^Saint James School of Medicine, USA; ^3^University of Medicine and Health Science, USA

## Abstract

The lifelong prevalence of bipolar disorder in adults, including subsyndromal forms, has increased over the years in the United States. By contrast, neurodegenerative diseases such as Parkinson's disease have demonstrated an age-related rise in prevalence. As the global population manages to live longer thanks to sociomedical developments, it is expected to observe a rise in the occurrence of comorbid neuropsychiatric disorders. Herein, we present the case and management regimen of a 51-year-old female patient with multiple comorbidities and in the presence of polypharmacy. She was diagnosed with Parkinson's disease and bipolar disorder type I alongside multiple comorbidities; her polypharmacy and medical history presented a significant clinical challenge in managing her condition. This case report focuses on the pharmacologic management of neuropsychiatric disorders titrated to this patient's particular needs, which were complicated by psychosis and comorbidities.

## 1. Introduction

While the subtypes are various, bipolar disorder, previously known as manic depression, encompasses an individual experiencing mood changes ranging from severe depression to a manic episode for varying periods of time. The American Psychiatric Association, Diagnostic and Statistical Manual of Mental Disorders, 5th edition, defines manic episodes as a distinct period of abnormally and persistently elevated, expansive, or irritable mood and abnormally and persistently increased goal-directed activity or energy, lasting at least one week and persisting most of the day, nearly every day (or any duration if hospitalization is necessary) [1]. A milder version of this is known as a hypomanic episode. In order to diagnose a patient as having bipolar disorder, presence of a manic or hypomanic episode on the patient's past medical history is warranted. The lifelong prevalence of bipolar disorder, including subsyndromal forms in the United States, has ranged from 0.9% to 2.1% [2]. Studies also indicate differences in lifetime prevalence estimates for bipolar disorder type I (1.0%), bipolar disorder type II (1.1%), and subthreshold bipolar disorders (2.4–4.7%) [3].

Treatment for bipolar disorder typically begins after first ruling out a drug-induced manic episode and is dependent on the current mood the patient is presenting, alongside any accompanying symptoms. This is to say, for an acute manic episode, the patient may be treated with antipsychotics, mood stabilizers such as lithium or valproate, or benzodiazepines. If the patient also presents with psychosis, agitation, aggression, or sleep disturbances, the treatment must be modified to suit the patient's needs. Likewise, in the presence of a depressive episode, treatment must be tailored to quickly stabilize the patient's condition. Common choices for initial therapy in newly diagnosed patients are atypical antipsychotics such as quetiapine or risperidone [4]. Choice of medication is heavily dependent on their side profiles and interactions with the current patient medications as well as their physical health and comorbidities.

Antipsychotics function through inhibition of dopaminergic pathways in the brain. These dopaminergic pathways include the mesolimbic, mesocortical, tuberoinfundibular, and nigrostriatal pathways. While inhibition of the mesolimbic and mesocortical pathways are the therapeutic targets of atypical antipsychotics, when it comes to the nigrostriatal pathway, the inhibition of D2 receptors in the striatum leads to disinhibition of GABA- and encephalin-containing striatal neurons. This is followed by disinhibition of the subthalamic nucleus leading to increased GABAergic inhibition of the thalamocortical projection [5], a mechanism that leads to the manifestation of extrapyramidal side effects such as drug-induced parkinsonism. While drug-induced parkinsonism may be clinically indistinguishable from Parkinson's disease (PD), it can be definitively diagnosed if the parkinsonism resolves within six months after stopping the offending agent [6].

Parkinson's disease (PD) is a degenerative disease that features a variety of signs and symptoms that include bradykinesia, postural instability, resting tremors, and neurobehavioral disorders (anxiety, depression, and dementia) [7]. Risk factors such as genetics, age, and toxins have been recorded and associated with the appearance of PD [8, 9]. PD has been described as a progressive neurodegenerative disorder caused mainly by a lack of dopamine in the brain [10, 11].

Within the etiology of PD, we must include dopamine, a neurotransmitter associated with functions such as movement, motivation, and memory. It is observed in depleted levels in the brain of patients with PD due to dopaminergic cell death. Dopamine absence in PD brains is one of the events leading to motor insufficiency and probably, a reason for the cognitive deficiency noted in some PD patients [11]. PD shows a prevalence in patients older than 65, reaching 1% in that population and close to 5% in individuals at 85 years of age and above [12]. The scenario in which PD presents in a clinically indistinguishable fashion as drug-induced parkinsonism may rise given that PD is due to low dopamine levels in the brain whereas drug-induced parkinsonism is through dopamine blockade in the brain, thus presenting very similar symptoms.

Treatment for PD generally aims to augment depleted dopamine stores in the Substantia Nigra, thus minimizing or eradicating symptoms and enhancing the quality of life [13]. As with bipolar disorder, initiation of treatment for PD is based on the severity of symptoms [12]. Pharmacologic therapy is aimed at reducing and even eliminating symptoms. The clinician should consider possible complications and nonmotor symptoms such as depression, anxiety, fatigue, cognitive impairment, autonomic dysfunction (e.g., orthostatic hypotension, constipation, incontinence, and dysphagia), sleep disturbances, or even the induction of a manic episode. While first-line treatment varies, it is commonly a trial of monoamine oxidase-B (MAO-B) inhibitors, dopamine agonists, carbidopa/levodopa, and anticholinergic agents. Other adjunctive therapies could be implemented depending on the effectiveness of the aforementioned treatment regimen or the appearance of new complications (e.g., refractory tremor, dyskinesia, and adherence of the patient to the current treatment) and the progression of the disease. [13].

## 2. Case Presentation

### 2.1. Chief Complaint

The patient was referred to the psychiatric department due to a current depressed and anxious state, suicidal ideations with a plan to cut her forearms, and homicidal ideations alongside auditory and visual hallucinations.

### 2.2. History of Present Illness

The patient is a 51-year-old female with a psychiatric medical history significant for Parkinson disease and bipolar disorder type I and a past medical history of generalized anxiety disorder, uncontrolled hypertension, hypertensive urgency, multiple cerebrovascular accidents, diabetes mellitus, fibromyalgia, hypoxia, hypoventilation associated with obesity syndrome, obstructive sleep apnea, chronic back pain, acute on chronic respiratory failure with hypercapnia, morbid obesity with a BMI > 50, and metabolic syndrome. Upon interview, the patient expressed experiencing auditory hallucinations with commanding voices to harm others. She was responding to internal stimuli and admitted to currently experiencing visual hallucinations consisting of different images each time. The patient mentioned she wanted to kill herself on the basis of being unable to handle her morbid state. On physical examination, the patient had persistence of a resting tremor prominent on the patient's left arm and hand as well as cogwheel rigidity and bradykinesia. Her reflexes were bilaterally diminished, and her strength was 4/5 on upper extremities. She was bedridden and unable to ambulate on the basis of morbid obesity and chronic back pain.

#### 2.2.1. Psychiatric Evaluation

On bedside examination, the patient was AOx4, her attitude was anxious but cooperative, she maintained good eye contact, appeared fairly groomed, and exhibited psychomotor retardation. Her speech was soft and slow, her mood was depressed, and her affect congruently sad. The patient's thought process was goal oriented and organized. She admitted to current hallucinations where she saw snakes and birds as well as moving shadows in an empty room. Her attention/concentration and recent memory were impaired as evidenced by inability to perform series of 7, list the days of the week backwards, or recall her last meal, respectively. She demonstrated insight into her condition, but her abstract reasoning and judgment were impaired. On a PHQ-9 questionnaire, she scored a 25, indicating severe depression in need of immediate intervention. On the young mania rating scale (YMRS), her score was 10 indicating that the patient was not manic at the time of examination.

#### 2.2.2. Psychiatric Past Medical History

The patient lost her mother in 1998 and recalls experiencing depressive symptoms and episodes of agitation, impulsivity, and racing thoughts since that event. In 2000, the patient was diagnosed with bipolar disorder type I, for which she was self-reportedly not adherent to the medication regimen attributed to growing tired of daily consumption of multiple pills. In 2002, the patient suffered an embolic stroke. Poststroke, the patient exhibited insomnia (sleeping 1.5 hrs. per night), agitation, racing thoughts, talkativeness, impulsivity, and aggressivity, which cooccurred with auditory and visual hallucinations which she denied having experience before said event. These symptoms are characteristic of a manic episode with psychotic features.

### 2.3. Medications and Management

Upon admission to our hospital, the patient's medications included lorazepam 1 mg bid, clonazepam 0.5 mg PRN q12h, risperidone 2 mg qd, mirtazapine 15 mg bid, duloxetine 60 mg bid, zolpidem 10 mg PRN, memantine 10 mg bid, pramipexole 0.5 mg tid, acetaminophen 325 mg bid, acetic acid otic 2% bid, mirabegron 25 mg qd, montelukast 10 mg qd, ocular lubricant in both eyes qid, pantoprazole 40 mg qd, prazosin 5 mg qd, pregabalin 100 mg tid, sucralfate 1 g qid, sulfasalazine 500 mg bid, albuterol 90 mcg/inh q6d, amlodipine 10 mg qd, aspirin 81 mg qd, baclofen 10 mg tid, butalbital/acetaminophen/caffeine (Fioricet oral capsule) PRN q8h, celecoxib 200 mg bid, docusate 100 mg qd, esomeprazole 40 mg qd, furosemide 40 mg qd, hydroxychloroquine 200 mg bid, levothyroxine 112 mcg qd, meclizine 25 mg tid, and fish oil 1000 mg qd.

During her stay in the hospital, the patient required a multidisciplinary approach to her care. The goal was to ensure that her physiological and psychiatric medical conditions were efficiently addressed. This rare case required a meticulous approach, given that some of the patient's medications such as benzodiazepines and mirtazapine have been associated with exacerbations of depressive mood and accentuation of anxiety, respectively, both of which may be present on a patient with BPD type I.

Differential diagnoses pivoted around mood disorders including bipolar disorder with mood congruent psychotic features, bipolar disorder 1, major depressive disorder with psychosis, major depressive disorder, brief psychotic episode, Parkinson's disease psychosis, and psychosis not otherwise specified. Final diagnosis was bipolar type I with melancholic features and mood-congruent psychotic features. Initial treatment approach was focused on stabilizing the patient through pharmacological management of her depressive mood with psychotic features.

Psychiatric stabilization was carried out by performing daily cognitive behavioral therapy (CBT) in combination with a quick cross-taper between quetiapine and risperidone over two days. We discontinued clonazepam, zolpidem, and duloxetine. Lorazepam and pramipexole were continued at the previously prescribed dosage and frequency. Mirtazapine 15 mg bid was discontinued, and mirtazapine 30 mg qhs was ordered. The patient received gradually decreasing doses of risperidone and gradually increasing doses of quetiapine until she was weaned off from risperidone entirely at which point, she was receiving 100 mg of quetiapine QHS. We initially decreased risperidone from 2 mg daily to 1 mg daily and administered quetiapine 50 mg qhs. The following day risperidone was discontinued, and quetiapine was increased to 100 mg qhs.

The patient was monitored with one-to-one personnel throughout her hospitalization to observe any changes in mood and thought content. Five days after initiating the patient on quetiapine, the dose was increased from 100 mg qhs to 150 mg qhs. We continued to monitor the patient; seven days after her first quetiapine dose, she was placed on quetiapine 200 mg qhs. Five days after initiation of quetiapine 200 mg qhs, the patient demonstrated significant improvement of her psychotic and depressive symptoms as evidenced by dissipation of visual and auditory hallucinations, a sleeping time averaging 6 h per night, and a PHQ-9 score of 9, indicative of mild depression.

At this point, the patient continued to exhibit symptoms characteristic of underlying Parkinson's disease. She was referred to a neurologic consult in order to address her condition and current Parkinson's disease medication regimen. She was also referred to family medicine as to address her polypharmacy. Further, the patient was referred for admission to a skilled nursing facility to best assist her with ambulation and medication adherence and provide a supportive and monitored environment.

## 3. Discussion

The patient was given a diagnosis of bipolar disorder type I with melancholic features and mood-congruent psychotic features given her past medical history of bipolar disorder type I and the current presentation of her symptoms. Her psychotic features included visual and auditory hallucinations which were mood congruent on the basis of the patient hearing commanding voices indicating her to harm herself or others. Whereas her melancholic features included psychomotor retardation, soft and slow speech, depressed mood, depressed affect, impaired attention and concentration, impaired judgment and abstraction, suicidal and homicidal ideations with the former presenting a clear plan, and a PHQ-9 score of 25. Treatment was initially tailored to address the patient's depressed mood with psychotic features which posed immediate danger to herself and others. Immediate placement of one-to-one personnel was warranted to help protect the patient from herself and better care for her current needs while also observing the efficiency of the medication regimen.

### 3.1. Treatment of Choice

When considering treatment of choice for patients in a psychotic state, there is a favorable trait in atypical antipsychotics that makes them preferable over first-generation antipsychotics; they have a lower incidence of extrapyramidal symptoms. In a patient with multiple comorbidities and medication nonadherence, this is particularly important to consider as to avoid worsening the patient's physical condition. Antipsychotic-induced extrapyramidal adverse effects are well recognized in the setting of first-generation antipsychotic drugs. However, research has shown that the creation and utilization of second-generation antipsychotics, with atypical mechanisms of action, significantly lower dopamine receptors' affinity, aimed at providing patients with an antipsychotic treatment which produces less extrapyramidal syndrome [14].

There have been reported cases of drug-induced parkinsonism which subside after elimination of the offending agent but recur months to years later or simply do not subside to begin with. “In most cases, such irreversible or temporarily reversible symptoms are felt to represent patients with early PD pathology that is too mild to manifest motor symptoms, and the dopamine receptor blockade “unmasks” their preclinical PD” [15].

Given the patient reported nonadherence to medication regimen, and her current presentation with parkinsonian features, it is appropriate to consider the possibility of an underlying early onset PD which may have been precipitated by initial antipsychotic medications previously prescribed. While this is a subject to be addressed by the neurological department, we still need to consider it as a factor regarding our choice of psychiatric pharmacotherapy.

It is generally accepted that second-generation antipsychotics (SGAs) are safer in patients with PD due to their lower D2 antagonism, but they also can cause extrapyramidal symptoms, although in lower rates in comparison with first-generation antipsychotics (FGAs) [16].

The Table 1, [17] provides a comparison of affinity to the dopamine-2 receptor across first- and second-generation antipsychotics, naming specific drugs in each generation. As with many treatment options, experts agree that when selecting an antipsychotic drug, the efficacy and adverse effects heavily depend upon the patient's age, gender, and previous medical conditions [18]. Given the multiple comorbidities present in our patient, we fared toward a drug that would be less likely to manifest significant extrapyramidal side effects. Our patient was utilizing risperidone, a second-generation antipsychotic, and we decided to discontinue it and use quetiapine instead as it was deemed most beneficial to our patient's current conditions which included psychosis, metabolic syndrome, parkinsonian features insomnia, and bipolar disorder with melancholic features.

#### 3.1.1. Risperidone versus Quetiapine

Risperidone and quetiapine belong to the second-generation antipsychotic drugs that have recently become the mainstay treatment regimen for schizophrenia and bipolar cases [19–21]. These drugs are considered superior in the management of psychosis in patients as compared to the erstwhile typical antipsychotic drug classes [22, 23]. Though using these second-generation drugs comes with better results in managing acute psychosis, there have not been detailed studies that have discussed the differences amongst the patients who have bipolar disorders. It has been demonstrated that the use of quetiapine, as opposed to risperidone, proved to be the ideal treatment regimen in managing concomitant bipolar disorder type 1 with melancholic features and mood congruent psychotic features [24].

#### 3.1.2. Addressing Bipolar Depression and Psychosis

A randomized study on quetiapine and risperidone in treating acute psychosis suggested better treatment outcomes when using quetiapine to treat psychosis [25]. The study observed that these drugs are almost similar, with marginal variations in their operational model. For instance, both agents in the two drugs were essential in improving social and cognitive functioning. The study intimates that in the positive and negative symptoms scale (PANSS) and the Global Impression-Severity scale (CGI-s), quetiapine is marginally superior in reducing PANSS psychopathology subscore. Notably, in the long term, quetiapine may be considered an effective treatment regimen for the patient compared to risperidone [26, 27]. Comparatively, the use of quetiapine has few side effects when used in relieving psychotic, manic, and depressive episodes in patients [28, 29].

The use of quetiapine as monotherapy has been chosen as first line treatment of acute bipolar depression and the prevention of mania/hypomania switching [30]. Furthermore, in a recent randomized trial, 1172 patients with a manic or depressive episode were stabilized with open-label quetiapine and then assigned to maintenance with quetiapine, lithium, or placebo; in the study, time to episode relapse was significantly longer in patients who were taking quetiapine as compared to those on lithium or placebo [31]. When it comes to duloxetine, while it may be useful for the treatment of chronic back pain and fibromyalgia, the effectiveness of antidepressants, whether prescribed as monotherapy or adjunctive therapy for bipolar depression is still unproven [32, 33]; it however does carry an inherent risk of precipitating mania in bipolar patients. The available data suggests that SNRIs can induce mood switching in patients with bipolar depression; the potential of duloxetine to induce manic or hypomanic symptoms cannot be disregarded [34].

Electroconvulsive therapy is a treatment of choice for patients with bipolar major depression who are severely ill (e.g., persistent suicidal ideation with a plan) and have not responded to multiple (e.g., three to five) pharmacotherapy trials [35]. This is a path to consider if our patient fails to improve after the prescribed management.

#### 3.1.3. Addressing Metabolic Syndrome

The use of quetiapine comes with a moderate risk of metabolic disorders [36–38], a crucial difference from risperidone which, due to its stronger affinity to the dopaminergic receptors, carries a significantly elevated risk of developing metabolic disturbances. Between the two atypical antipsychotics, it is evident that the use of quetiapine comes with fewer side effects [38] supporting the choice of quetiapine over risperidone in our patient.

#### 3.1.4. Addressing Parkinsonian Features

It is unknown when the patient started exhibiting parkinsonian symptoms, which may have been secondary to medication, a diagnosis of early onset Parkinson's disease, however, is better justified given self-reported medication nonadherence for various years, with persistence of a resting tremor prominent on the patient's left arm and hand as well as cogwheel rigidity and bradykinesia which were reportedly present at the time of admission to the skilled nursing facility. At the time of arrival at our hospital, the patient had already been diagnosed with Parkinson's disease. Treating the patient with a drug carrying high risk for EPS such as risperidone in the presence of her PD diagnosis would prove detrimental to the patient's health through exacerbation of her parkinsonian features [39].

#### 3.1.5. Addressing Insomnia

Insomnia impairs cognitive and physical functioning and is associated with a wide range of impaired daytime functions across several emotional, social, and physical domains [40]. Although benzodiazepines improve short-term sleep outcomes, they have significant adverse effects and may be addictive [41]. The patient presented with a sleep time averaging 90 minutes per night, despite having hypnotic medications such as zolpidem and benzodiazepines as part of her prescription medications. Of note, medication nonadherence may also have contributed to her insomnia. We discontinued clonazepam and zolpidem. Given that our patient was already presenting with comorbid respiratory impairment, maintaining two benzodiazepines and a nonbenzodiazepine hypnotic carried a high risk for respiratory depression and subsequent failure [42]. Lorazepam was continued at the previously prescribed dosage and frequency. Mirtazapine 15 mg bid was discontinued, and mirtazapine 30 mg qhs was ordered, while it had been previously prescribed to address her fibromyalgia and depression; its hypnotic side profile was utilized to best assist our patient through a change in medication timing. Given their action on histamine receptors, second-generation antipsychotics commonly cause sedation. In addition to this effect, quetiapine also exhibits sleep latency-enhancing properties, attributable to its serotonergic action [43] making it a suitable choice of medication for our patient's profile.

#### 3.1.6. Quick Cross Taper of Quetiapine and Risperidone

Once it was determined that the discontinuation of risperidone and prescription of quetiapine was warranted, we opted for a method which allowed for us to minimize side effects associated with abrupt discontinuation of antipsychotics. Research has found that at times, switching antipsychotics is necessary to address instances of adverse effects, nonresponse, or partial response. The pharmacodynamics and pharmacokinetics of the drug in use and the alternative drug are considered alongside the clinical situation and the history of response to other antipsychotics to determine the appropriateness of the cross taper [29].

Quetiapine has a high receptor affinity to muscarinic receptors [44]. Risperidone, on the other hand, has little to no affinity to muscarinic receptors. If caution is not maintained when switching from quetiapine to risperidone, a transient anticholinergic rebound may be prompted, leading the patient to experience anxiety, agitation, or insomnia [44]. As a matter of fact, switching from quetiapine, which has a strong binding affinity to the H1 receptor, to risperidone that has a weaker or no binding to the H1 receptor is associated with a rebound, restlessness, insomnia, and agitation. Similarly, when switching from risperidone, which is a tighter D2 binding agent, to quetiapine, a looser D2 binding agent, worsening of symptoms of mania, psychosis, or agitation/aggression may occur [44], and these side effects need to be monitored, often warranting one to one placement in order to carefully monitor patients for changes in mood or behavior. Risperidone also has a strong binding affinity to 5-HT2A while quetiapine has a weak binding affinity to 5-HT2A, abruptly switching from risperidone to quetiapine may cause a serotonin syndrome or neuroleptic malignant syndrome (NMS) symptom [44]. The potential for rebound or withdrawal phenomena is high when the drugs are switched abruptly. Short cross tapers between risperidone and quetiapine avoid fast or abrupt switching that would have been problematic since the two drugs have varying receptor affinities [44].

## 4. Conclusion

Switching from risperidone to quetiapine was deemed more effective compared to other cross tapers because quetiapine can be used as monotherapy, thus minimizing polypharmacy [45]. This approach was found to be very beneficial to our patient. Most clinicians prescribe cross tapering with quetiapine rather than other second-generation antipsychotics [45]. Advantages of cross-tapering with quetiapine over alternatives include the lower likelihood of resistance to treatment, shorter duration of hospitalization, and lesser risk of adverse events. Cross-tapering between risperidone and quetiapine is unlikely to cause withdrawal symptoms given their comparative effect on various receptors [46, 47]. When faced with complicated case presentations in the presence of polypharmacy, it falls to the clinician the responsibility of carefully reassessing the patient's needs and current medications. Furthermore, if more efficient medications are available to best assist one or more of the presenting issues, monotherapy should be considered over utilizing a plethora of medications which may exacerbate the patient's medical issues through either nonadherence to medication due to polypharmacy or even side effects or drug interactions which may iatrogenically hurt the patient.

## Figures and Tables

**Table 1:** [17] First- and second-generation antipsychotics and D2 antagonism.

Antagonistic D2 effect	First-generation antipsychotics	Second-generation antipsychotics
Low	Chlorpromazine Levomepromazine Thioridazine	Clozapine Quetiapine
Intermediate	Trifluoperazine Perphenazine	Olanzapine
High	Haloperidol Fluphenazine Flupentixol	Risperidone Ziprasidone Aripiprazole (possible D2 agonism)

Table 1 is reproduced from Divac et al. 2015 [under the Creative Commons [Attribution License/public domain]].

## Data Availability

Data is included within the article.
